# Anaemia and pregnancy: Anaesthetic implications

**DOI:** 10.4103/0019-5049.71026

**Published:** 2010

**Authors:** Anju Grewal

**Affiliations:** Department of Anaesthesiology, Dayanand Medical College and Hospital, Ludhiana, Punjab, India

**Keywords:** Anaemia, anaesthetic considerations, compensatory mechanisms, pregnancy

## Abstract

Anaemia in pregnancy defined as haemoglobin (Hb) level of < 10 gm/dL, is a qualitative or quantitative deficiency of Hb or red blood cells in circulation resulting in reduced oxygen (O2)- carrying capacity of the blood. Compensatory mechanisms in the form of increase in cardiac output (CO), PaO_2_, 2,3 diphosphoglycerate levels, rightward shift in the oxygen dissociation curve (ODC), decrease in blood viscosity and release of renal erythropoietin, get activated to variable degrees to maintain tissue oxygenation and offset the decreases in arterial O_2_ content. Parturients with concomitant medical diseases or those with acute ongoing blood losses may get decompensated, leading to serious consequences like right heart failure, angina or tissue hypoxemia in severe anaemia. Preoperative evaluation is aimed at assessing the severity and cause of anaemia. The concept of an acceptable Hb level varies with the underlying medical condition, extent of physiological compensation, the threat of bleeding and ongoing blood losses. The main anaesthetic considerations are to minimize factors interfering with O_2_ delivery, prevent any increase in oxygen consumption and to optimize the partial pressure of O_2_ in the arterial blood. Both general anaesthesia and regional anaesthesia can be employed judiciously. Monitoring should focus mainly on the adequacy of perfusion and oxygenation of vital organs. Hypoxia, hyperventilation, hypothermia, acidosis and other conditions that shift the ODC to left should be avoided. Any decrease in CO should be averted and aggressively treated.

## INTRODUCTION

WHO estimates indicate a 65-75% prevalence of anaemia in pregnant women in India.[1,2] Nearly half of the global maternal deaths due to anaemia occur in South Asian countries with 80% of these being contributed by India.[1,3]

## DEFINITION OF ANAEMIA

Anaemia is a qualitative or quantitative deficiency of Hb or red blood cells (RBC) in circulation resulting in a reduced oxygen (O_2_)-carrying capacity of the blood to organs and tissues.[4] Anaemia in pregnancy is defined as an Hb concentration of < 11 gm/dL or a haematocrit < 0.33 in first and third trimesters, while in the second trimester a fall of 0.5 gm/dL is adjusted for an increase in plasma volume and a value of 10.5 gm/dL is used.[5,6] However, in India and most of the other developing countries a lower limit of 10 gm/dL is often accepted.[7]

## CLASSIFICATION OF ANAEMIA

Anaemia during pregnancy may be classified based on etiology as

- Physiological anaemia of pregnancy
- Acquired:
  - Nutritional- Iron deficiency, folate deficiency, B-12 deficiency, etc.
  - Infections- Malaria, hookworm infestation, etc
  - Haemorrhagic- Acute or chronic blood loss
  - Bone marrow suppression- Aplastic anaemia, drugs, etc.
  - Renal disease
- Genetic - haemoglobinopathies – sickle cell disease, thalassaemia, etc

Anaemia in pregnancy can also be classified as mild, moderate or severe, with WHO classifying mild anaemia as Hb level of 10.0-10.9 gm/dL, moderate anaemia as 7-9.9 gm/dL and < 7gm/dL as severe anaemia.[8]

## PHYSIOLOGICAL HAEMATOLOGICAL CHANGES IN PREGNANCY PERTINENT TO ANAEMIA

Maternal blood volume begins to increase early at 6^th^ week and continues to rise by 45-50% till 34 weeks of gestation, returning to normal by 10-14 days postpartum.[9–13] This adaptive physiological hypervolemia helps to maintain blood pressure in presence of decreased vascular tone[9,14,15], facilitates maternal and fetal exchange of respiratory gases, nutrients and metabolites and protects the mother from hypotension, by reducing the risks associated with haemorrhage at delivery.[10]

Increased fetal and maternal production of estrogen and progesterone contribute to the rise in plasma volume.[10,16] Progesterone enhances aldosterone production. Both esterogen and aldosterone increase plasma renin activity, enhancing renal sodium absorption to 900 mEq and water retention to 8.5 L approximately, via the renin-angiotensinaldosterone system.[10,17] The concentration of plasma adrenomedullin, a potent vasodilating peptide, rises during pregnancy, and correlates significantly with blood volume.[10,18]

RBC volume decreases during the first 8 weeks, increases to the prepregnancy level by 16 weeks, and undergoes a further rise to 30% above the prepregnancy volume at term.[9,10,12,14,19] Elevated erythropoietin concentration[9,20] and the erythropoietin effects of progesterone, prolactin and placental lactogen[9] result in an increase in RBC volume.[9,14]

Hence the plasma volume expansion increase exceeds the rise in RBC volume, resulting in haemodilution and consequent physiological anaemia of pregnancy,[9–14] with an average Hb and haematocrit of 11.6 gm/dL and 35.5%, respectively.[21] This represents a 15% decrease from prepregnancy levels.[9] The decrease in blood viscosity from the lower haematocrit reduces resistance to blood flow, as a compensatory mechanism.[10] However, if the Hb concentration falls < 10 gm/dL, other causes of anaemia should be considered.[9]

## PATHOPHYSIOLOGY OF ANAEMIA

The anaesthetic implications of anaemia in pregnancy stem from the adverse effects of decreased tissue O_2_ delivery. Let us briefly review the normal and compensatory O_2_ delivery mechanisms in anaemia.

Oxygen is carried in the blood in two forms as:

- Physical solution in plasma (dissolved form)
- Reversible chemical combination with haemoglobin (Oxyhaemoglobin)

Arterial blood contains only 0.3 mL of O_2_, in each 100 mL of blood at a PO_2_ of 100 mm Hg and temperature of 37°C.[22] This small quantity reflects tension of O_2_ in the blood and acts as a pathway for the supply of O_2_ to Hb and for the transfer of O_2_ to cells.

Majority of the O_2_ carried in blood is in combination with Hb. As blood leaves the lung, Hb reversibly binds to four molecules of O_2_ which equals to 1.37-1.39 mL/g of Hb.[22,23]

Therefore, the O_2_ content of the blood is the quantity of O_2_ contained in the red cell added to the quantity dissolved in plasma, defined as the volume of O_2_ in milliliters carried in 1 dL of blood. It is calculated from the equation:[22]

$$\;{\mathrm{CaO}}_{2}=\mathrm{Hb}\;\times \;1.37\;\times \;{\mathrm{SaO}}_{2}\;+\;0.0034\;\times \;{\mathrm{PaO}}_{2}\;\mathrm{mm}\;\mathrm{Hg}$$

[a-arterial sample; CaO_2_- arterial O_2_ content in mL/dL of blood, Hb-concentration of Hb in gm/dL; 1.37 is the volume of O_2_ in milliliters carried by 1 g of fully saturated Hb; SaO_2_-fractional Hb saturation defined as the ratio of oxyHb to total Hb, hence SaO_2_ =HbO_2_ / (HbO_2_ + reducedHb + methHb + COHb); 0.0034 are the solubility coefficients of O_2_ in plasma (mL of O_2_/dL plasma in mm Hg); PaO_2_-arterial O_2_ tension measured in mm Hg.]

Hence, the O_2_ content of the blood where PO_2_ is 100 mm Hg, SaO_2_ is normal and Hb concentration is 15 gm/ dL, is 20 ml. In anaemia when Hb concentration falls by 50% (7.5 gm/dL), O_2_ content decreases to 10 ml/dL. Thus, Hb and SaO_2_ are the primary determinants of arterial O_2_ content.[22,23]

This total quantity of O_2_ in arterial blood delivered to tissues is a function of cardiac output (CO). Therefore, Oxygen delivery= CaO_2_ × Cardiac Index × 10 mL/ min/m^2^

Whenever anaemia occurs, i.e., CaO_2_ decreases, CO increases as a compensatory mechanism to maintain O_2_ delivery to tissues.[22]

Oxygen consumption (VO_2_), an important determinant of adequacy of tissue oxygenation, is determined primarily by CO and arterio-venous O_2_ content difference C (a-v)O_2_.[7,22,23]

$$\;{\mathrm{VO}}_{2}\;=\;\mathrm{CO}\;\times \;\left({\mathrm{CaO}}_{2}\;-\;{\mathrm{CvO}}_{2}\right)\;\times \;10\;=\;\mathrm{CO}\;\times \;\mathrm{Hb}\;\times \;1.37\;\times \;\left({\mathrm{SaO}}_{2}\;-\;{\mathrm{SvO}}_{2}\right)\;=\;230-250\;\mathrm{mL}\;O_{2}/\min$$

Hence, O_2_ extraction = 250/1000 mL O_2_ = 25%

In chronic anaemia, CO increases to maintain a constant arterial venous O_2_ content difference and the O_2_ extraction ratio. However, in an anaemic parturient, whenever the O_2_ demand rises acutely, the SvO_2_ falls to enhance O_2_ extraction along with a further increase in CO as compensatory mechanisms. Acute cardiac failure may result due to excessive strain on myocardium, hence increases in CO should be < 10 L/min.[23]

**Figure 1:**
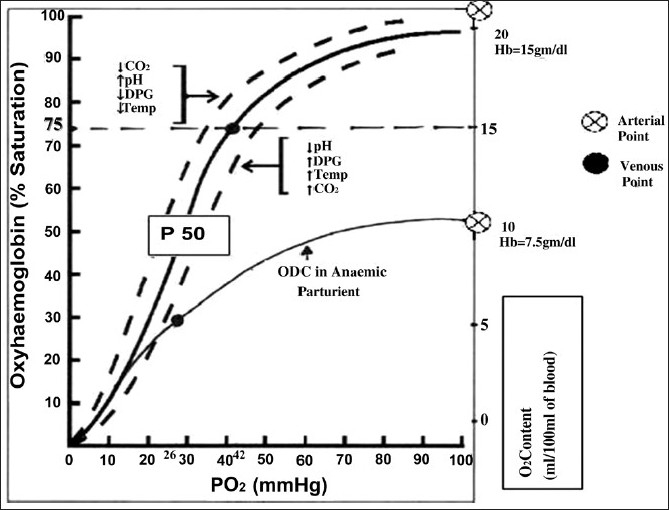
Oxygen dissociation curve

Adequacy of tissue oxygenation (tissue PO_2_) is judged by mixed venous PO_2_ (PvO_2_).[22,23] Normal values of PvO_2_ is 35-45 mm Hg (5-6 KPa), which corresponds to a normal mixed venous O_2_ content difference CvO_2_ of 12-15 vol% and a mixed venous Hb saturation SvO_2_ of 72-78%.[23–25] Thus, we can deduct that as PvO_2_ falls from 40 - 47 in normal patients to 26 mm Hg in severe anaemia [Figure 1], tissue hypoxia may occur. In an attempt to prevent tissue hypoxia the body decreases the amount of O_2_ released per 100 mL of blood and effectively moves the mixed venous point, by increasing the tissue blood flow and CO.[7,22,23] The cardiovascular system of the patient should be healthy enough to compensate for increases in CO. Hence, the risk of anaemia will depend both on the magnitude of fall in tissue O_2_ content and on the nature and severity of coexisting medical diseases.

Apart from an increase in CO and O_2_ extraction, the O_2_ delivery is greatly affected by the relationship between the saturation of Hb with O_2_ (SaO_2_) and the partial pressure of O_2_ in the blood, best described by the oxygen dissociation curve (ODC)[23] [Figure 1]. The Hb molecule has specific characteristics which allows the oxygenation of one subunit of Hb molecule to facilitate 300 times greater O_2_ affinity of the other subunits. Similarly release of O_2_ from the first Hb subunit will facilitate the release of further O_2_. “This enhancement of O_2_ uptake and release is the cause of the sigmoidal shape of ODC.”[22] The sigmoid shape is important as the rapid descent allows a large fraction of O_2_ to be released to the tissues with a modest drop in the partial pressure of O_2_ [22,23] [Figure 1]. The partial pressure of O_2_ in the blood at which the Hb is 50% saturated, known as P_50_ is 26.6 mm Hg at a pH of 7.4.[22]

The P_50_ is a conventional measure of Hb affinity for O_2_. Increased temperature and rise in hydrogen ion [H^+^] and 2,3- diphosphoglycerate (DPG) concentrations reduce the affinity of Hb for O_2_, leading to an increase in the P_50_ and rightward shift of the curve thus facilitating the unloading of O_2_ at peripheral tissues. A decrease in P_50_ indicates a left shift in the ODC and an increased affinity of Hb for O_2_, so that a lowerthan- normal O_2_ tension saturates Hb in the lung and the subsequent release of O_2_ to the tissues occurs at a lower-than-normal capillary O_2_ tension[22,23] [Figure 1].

Changes in 2,3-DPG are most important and often seen in chronic anaemia especially sickle cell anaemia, and chronic hypoxemia. 2,3-DPG is a metabolic product of anaerobic metabolism with a normal intraerythrocyte concentration of 15 μmol/gm Hb. It is able to bind with β-chains when Hb is deoxygenated, thus increasing O_2_ availability[7,22,23,26–28].

To summarize, in an anaemic pregnant patient various compensatory mechanisms get activated:[7,22,23,24,28]

1. Increase in CO
2. Rightward shift of ODC
3. Decrease in blood viscosity
4. Increase in 2,3-DPG concentration in RBC
5. Release of renal erythropoietin leading to stimulation of erythroid precursors in bone marrow

Thus, though tissue oxygenation is not impaired during physiological, or chronic anaemia as a result of compensatory mechanisms, these may be compromised in severe or acute onset anaemia leading to serious consequences like right heart failure, angina, tissue hypoxemia, etc.[7,28]

## ANAESTHETIC CONSIDERATIONS

### Preoperative assessment

Clinical assessment should focus at assessment of the cause, type and severity of anaemia and adequacy of compensatory mechanisms.[7,28]

- History suggestive of poor tissue perfusion can manifest as tiredness, easy fatiguability in mild anaemia to breathlessnesss/dyspnea, palpitations, angina in moderate to severe anaemia.[7,28]
- Signs of high CO like tachycardia, wide pulse pressure and systolic ejection murmur are essential for planning the mode of anaesthetic management.
- Investigations should include a complete haemogram, reticulocyte count, peripheral smears and blood grouping. Other investigations include stool and urine analysis, ESR, blood urea nitrogen levels, S. creatinine, bilirubin levels, S. proteins S. Iron, total iron-binding capacity, B12 and folate levels, Hb electrophoresis and ECG for any evidence of myocardial ischaemia, etc.[7,28]

### Minimal acceptable level of Hb and need for preoperative transfusion

A ‘minimum acceptable haemoglobin level’ does not exist.[29] A healthy myocardium compensates for the low Hb or Hct levels (7-8 gm/dL of Hb or 21-24% Hct) in order to optimize O_2_ delivery. In patients with overt or silent episodes of myocardial ischaemia (diabetic parturient), a level of < 10 gm/dL carries risk of decompensation.[29,30]

Many task force guidelines recommend that RBC transfusions should not be dictated by a single Hb “trigger”; instead, it should be based on the patient’s needs and risks of developing complications of inadequate oxygenation.[31–34] The decision to perform RBC transfusion should be made on both clinical and haematological grounds. Transfusion is rarely indicated in the stable patient when Hb is > 10 gm/dL and is almost always indicated when < 6 gm/dL.[32,33] If the Hb is < 7-8 gm/dL in labour or in the postpartum period, the decision to transfuse should be made on an informed basis according to the symptoms, coexisting medical conditions, continuing blood loss or threat of bleeding. There is little evidence of the benefit of blood transfusion in asymptomatic parturients.[30,33,34]

Benefits from replenishing O_2_-carrying capacity by transfusion must always be balanced against transfusion-associated risks like pulmonary oedema, immune suppression, etc.[35,36] In a large randomized controlled trial (RCT), Hebert established that there was no difference in mortality rates between restrictive and liberal transfusion strategies in noncardiac, critically ill patients who were able to tolerate lower levels of Hb.[36,37] Reiles and Linden[38] indicated in a study that the maintenance of a higher Hb concentration with RBC transfusion in an attempt to increase tissue O_2_ delivery is not associated with clinical benefit, as transfusion-related increased blood viscosity can result in a reduction in blood flow and incipient cardiac failure. Also the storage process affects the ability of RBCs to transport and deliver O_2_ to the tissues, due to decreases in erythrocyte concentrations of 2,3-DPG to 1 *μ*mol/g of Hb or less at 21 days of storage.[27,39] This point, however, remains controversial.[27]

If transfusion is necessary for severe cases of chronic anaemia, leukoreduced red cells given carefully under strict monitoring have beneficial effects.[38] Further research should be done to evaluate symptomatic transfusion strategy to a Hb-based strategy on the outcome of high-risk parturients.

### Choice of anaesthesia

Choice of anaesthesia will depend on the severity and type of anaemia, extent of physiological compensation, concomitant medical conditions, type and nature of procedure and anticipated blood loss. The main anaesthetic considerations in chronic anaemia are to minimize factors interfering with O_2_ delivery, prevent any increase in O_2_ consumption and to optimize the partial pressure of O_2_ in the arterial blood. The following measures need to be diligently adhered to in the perioperative period, while giving either General Anaesthesia or Regional anaesthesia:[7,10,28]

- Avoidance of hypoxia
  - Preoxygenation is mandatory with 100% O_2_.
  - Oxygen supplementation should be given in the peri- and postoperative period.
  - Maintenance of airway is important to prevent fall in FiO_2_ due to airway obstruction, difficult intubation, etc. Hence measures and expertise to secure a definitive airway should be available immediately.
  - Spontaneous ventilation technique is suitable only for short procedures. High FiO_2_ (40-50%) is administered to overcome effects of hypoventilation. High concentration of volatile agents depresses both the myocardium as well as ventilation resulting in an undesirable decrease in O_2_ flux.
  - Aggressively treat and avoid conditions that increase the O_2_ demands like fever, shivering, acute massive blood losses leading to an acute drop of Hb below 7 gm/dL.
  - Nitrous oxide should be used cautiously in patients with folate and Vitamin B-12 deficiency.
- Minimize drug-induced decreases in CO
  - Intravenous induction of anaesthesia should be slowly titrated to prevent precipitous fall in CO.
  - Careful positioning of the patient to minimize position associated volume shifts.
  - Mild tachycardia and wide pulse pressure may be physiological and should not be confused with light anaesthesia
- Factors leading to left shift of ODC should be avoided
  - Avoid hyperventilation to minimize respiratory alkalosis. Hypocapnia also decreases CO. Maintain normocapnia.
  - Hypothermia should be avoided –
    - Take all measures to ensure normal core body temperatures
    - IV fluids and blood products if any should be warmed
- Monitoring should be aimed at assessing the adequacy of perfusion and oxygenation of vital organs.[34] It should include routine monitors like ECG, NIBP, EtCO_2_, Temperature monitoring, Pulse oximetry, urine output and may include CVP, invasive arterial blood pressure monitoring, ABG analysis and measurement of mixed venous PvO_2_ in severe anaemia wherein major blood losses are anticipated like in placenta previa or acccreta etc. Serial Hb and Haematocrit values can guide us to monitor ongoing blood losses.[27]
- Regional anaesthesia is preferred for peripheral limb surgery as they are associated with reduced blood loss.
- Central neuraxial blocks can be judiciously employed using either a low-dose spinal anaesthesia along with adjuvants or an intermittent dosing, continuous epidural. These are advantageous in providing good analgesia, ability to provide supplemental O_2_, and decreased blood loss with stable haemodynamics. However, central neuraxial blocks are fraught with imminent dangers of hypotension, haemodilution and subsequent heart failure or pulmonary oedema on the return of vascular tone. It is advisable to use vasoconstrictors to sustain blood pressure.[7,28] Regional anaesthesia can also be implicated in the worsening of symptoms of subacute degeneration of spinal cord and hence should be avoided in parturients with overt Vitamin B_12_ deficiencies with neurological symptoms.[7,28]

### Special situations

#### Sickle cell disease

Sickle cell disease is a congenital haemoglobinopathy.[40] Parturients with sickle cell anaemia have an increased incidence of preterm labour, placental abruption, placenta previa and hypertensive disorders of pregnancy.[41]

In Hb S, valine is substituted for glutamic acid at the 6^th^ amino acid in the β-chains. This causes the Hb molecules to aggregate when deoxygenated.[24,40] HbS begins to aggregate at a PO_2_ of < 50 mm Hg, with all getting aggregated at a PO_2_ of 23 mm Hg.[24,42]

Dehydration, hypotension, hypothermia, acidosis and a high concentration of HbS, predispose the pregnant patient to sickling.[27] Sickle cell anaemia is a chronic anaemia. Marked ventricular hypertrophy secondary to increased CO, may lead to a deterioration in ventricular diastolic function.[24,43]

Blood transfusions need to be given only when they are specifically indicated (e.g., severe anaemia, hypoxemia, preeclampsia, septicemia, renal failure, acute chest pain syndrome, anticipated surgery, aplastic crisis). The goals of transfusion are to achieve a Hb concentration > 8 gm/dL and HbA > 40% of the total Hb.[24]

Anaesthetic management aims at avoidance of hypoxemia, hypovolemia, hypothermia and acidosis along with provision of good analgesia.[24,40] Both neuraxial and general anaesthesia are acceptable.[44]

#### Thalassaemia

Thalassaemias are a diverse group of microcytic, haemolytic anaemias wherein there is a reduced synthesis of one or more of the polypeptide globin chains.[24]

Advances in the management of β-thalassaemia major by extensive blood transfusions and chelation therapy have prolonged life expectancy.[45] Higher transfusion requirements in pregnancy worsen haemosiderosis and cardiac failure.[24]

The major anaesthetic considerations are due to chronic anaemia with resultant tissue hypoxia, multiple transfusions leading to increased iron load especially in the myocardial cells and concomitant difficult airway. Both general anaesthesia and central neuraxial anaesthesia[45] can be safely administered for cesarean delivery after estimation of the platelet count and after excluding history of spontaneous haemorrhage.[24]

## CONCLUSION

The anaesthetic implications of anaemia in pregnancy are based on the understanding of the normal and compensatory mechanisms that optimize tissue oxygenation. The main aim is to maintain a fine balance between the compensatory mechanisms and adequate tissue oxygenation in these parturients. Both regional and general anaesthesia can be used judiciously. Monitoring should aim at assessing the adequacy of perfusion and oxygenation and the magnitude of ongoing losses. Deleterious effects of chronic tissue hypoxemia along with threat of major blood losses in the perioperative period need to be anticipated and treated adequately.
